# Key indicators for appraising adolescent sexual and reproductive health in South Asia: international expert consensus exercise using the Delphi technique

**DOI:** 10.1080/16549716.2020.1830555

**Published:** 2020-10-20

**Authors:** Furqan Ahmed, Ghufran Ahmad, Tilman Brand, Hajo Zeeb

**Affiliations:** a Department of Prevention and Evaluation, Leibniz Institute for Prevention Research and Epidemiology – BIPS, Bremen, Germany; b NUST Business School (NBS), National University of Sciences and Technology (NUST), Islamabad, Pakistan; c Health Sciences Bremen, University of Bremen, Bremen, Germany

**Keywords:** Adolescent sexual and reproductive health, Delphi technique, indicator prioritization

## Abstract

**Background:**

There is a need for an accurate assessment of the patterns and determinants of sexual and reproductive health in South Asia owing to high fertility rates and high incidence of unplanned pregnancy among adolescents. Health indicator sets, with a wide range of health dimensions, also support in formulating evidence-based policies. For attaining this, indicators should be developed and prioritized based on consensus and relevance.

**Objective:**

This study aimed to develop a comprehensive list of adolescent sexual and reproductive health (ASRH) key indicators for South Asia through systematic participatory expert consultation exercise using the Delphi technique.

**Methods:**

Experts were invited to two rounds of an indicator rating exercise and a third round to discuss the results in a broader regional perspective. A list of nine indicator categories, including 41 adolescent health indicators, was rated by the expert panel. Prioritization was based on mean Likert scores while consensus was established using Kendall’s W.

**Results:**

24, 16 and six experts participated in the first, second and third round, respectively. Out of the nine indicator categories, demographics, reproductive health, violence, and nutrition were ranked high in relevance by the expert panel. Experts had a strong consensus on the relevance of parental control and connection, and behavioral indicators while there was moderate consensus on the relevance of nutrition, infectious disease, and mortality indicators.

**Conclusion:**

As far as we know, this is the first study that employs the Delphi technique for prioritizing ASRH indicators for South Asia. Engaging a diverse group of experts, using an online platform, we developed a comprehensive list of key indicators for appraising ASRH relevant to South Asia based on expert panel consensus and recommendations. Our results also highlight that there is a need for developing a region-specific prioritized list of indicators which might assist in identifying regional health needs.

## Background

During adolescence (10–19 years), rapid changes in social development, health, and wellbeing take place. Due to these reasons, a high priority has been given to the health, social development, and well-being of the adolescent population. The International Conference on Population and Development (1994) called on countries to educate and promote adolescent sexual and reproductive health (ASRH) [1]. Unfortunately, progress has been slow owing to misconceptions, organized community resistance regarding sexuality education, and implementation barriers in many regions of the world.

Globally, the foremost causes of death among adolescent girls are suicide, complications during childbirth and pregnancy [2]. Worldwide, almost 16 million adolescent girls aged between 15 and 19 years and 2.5 million under the age of 16 years give birth annually [3]. Unplanned and early pregnancy not only carries health risks for the young mother and child but also may be detrimental to the social, physiological, and psychological development of young girls [3]. According to the WHO, almost 1 million girls aged < 15 years give birth each year and 3 million girls aged 15–19 undergo unsafe abortions due to unplanned pregnancy [3]. Reports and literature reflect ignorance or destructive cultural norms in some countries: for example, two out of three girls in Low and Middle-Income Countries (LMICs) were unaware of what was happening to them when they first started menstruating [4–7]; the condom use in young people (15–24 years) at last high-risk sex in the previous year (non-marital, non-cohabiting sexual partner) was less than 50% [8]; and almost 50% of the girls worldwide believe that a husband or partner is justified in hitting or beating his wife or partner in certain circumstances [9].

South Asia consists of eight countries: Afghanistan, Bangladesh, Bhutan, India, Maldives, Nepal, Pakistan, and Sri Lanka. According to the World Bank, South Asia had a population of 1788.38 million in 2019, which is almost 20% of the total world population [2]. In LMICs, adolescents and young adults face many challenges due to poverty, inequality, and marginalization which adversely affect their mental and physical health and overall well-being [10]. In 2017, the annual population growth was 1.2% [2], the fertility rate was 2.4 births per woman while among adolescents (15–19 years) the fertility rate was 25.6 births per 1000 women in South Asia [2]. Males from 10 to 24 years of age are about 28.7% of the total male population and females of the same age are about 27.7% of the total female population in South Asia [2]. As almost one-third of the population in the region is an adolescent or late adolescent, there is a need to assess their health situation and circumstances affecting their health. Accurately assessing the situation, patterns, and determinants of reproductive health are critical due to the high fertility rates and incidence of unplanned pregnancies among adolescents in the region.

Reliable health indicators are essential for trustworthy and sound information on the health situation, patterns, and trends to help develop appropriate responses at the national, regional, or global level [11–14]. For assessing the health situation of a region, health indicators play a vital role in providing an overview of key gaps and health disparities [11–14]. Health indicators and data also guide in determining the priorities for investments in health, measure the health of a population, determine inequalities for different population segments, and to ascertain whether performance expectations are met or not in health [11–14]. Health indicator sets with a wide range of health dimensions also support in evidence-based policy synthesis [15]. For attaining this, indicators should be developed and prioritized based on consensus and relevance [14,16]. Understanding and priorities vary among stakeholders, experts, and policymakers regarding what connotes a perfect indicator [17,18]. This variation can also be seen among different regions of the world which can be attributed to regional disparities in health literacy, health priorities, health determinants, cultural norms, and demographics [19].

International experts and organizations (e.g. UNICEF and WHO) have developed numerous constructs, definitions, and prioritizations of adolescent health indicators [11–13,20]. These efforts have resulted in the development of a comprehensive adolescent health indicator list encompassing multiple health and social developmental aspects [11–13,20]. Most of the adolescent health indicator lists available are commonly used for LMICs but are not region-specific [11,12,21,22]. However, literature suggests that social determinants and contextual factors strongly affect reproductive health outcomes and service utilization, especially among the adolescent population [23–25]. Consequently, adolescent health determinants of social development, health and wellbeing differ widely across different geographical regions. Regional sets of health indicators might be able to provide a better insight into health needs, patterns, perspective, and data gaps regarding specific populations.

There are multiple techniques and methodologies to select indicators. Two of the methods for prioritizing indicators are: 1. Academics and researchers simply choosing indicators, which they give credence to as the most relevant indicators, and 2. Participatory methodologies for identifying and prioritizing indicators [26,27]. Using the second approach increases the chances that the prioritized indicators will be deemed more credible and relevant [28]. For this purpose, we developed a comprehensive list of ASRH key indicators for South Asia through a systematic participatory expert consultation exercise.

## Methods

### Modified Delphi technique

Delphi technique is a method that aids in structuring a group communication process and allows the participants to deal with an intricate problem as a group [29–32]. Delphi technique has numerous advantages including simplicity of implementation, collecting opinions of a vast array of participants with distinct expertise located in various geographical locations, while ensuring anonymity during the process [29–32]. For this purpose, a multidisciplinary panel of experts was identified and engaged for prioritizing and selecting ASRH indicators for South Asia. We relied on an online survey with a group of international experts, and for this, the Delphi technique was appropriate as the experts do not have to meet face-to-face during the Delphi process [30]. We conducted three Delphi rounds. The first two rounds provided the experts with the opportunity to rate indicators and were conducted in anonymity. Many modifications to the original Delphi have been used for conducting consensus exercise [31,32]. The third group discussion round was the main modification to the original Delphi technique where we invited the experts to discuss the results in a wider regional perspective.

### Literature review and initial list of indicators

The initial list of indicators was developed by a thorough literature search for existing lists of key ASRH indicators. A WHO report of a technical consultation on indicators for adolescent health was determined as the main source of this list. From this report, 27 adolescent health indicators on health determinants, outcomes, and service delivery – grouped within nine categories – were used for rating in the first round [11,12].

### Expert inclusion criteria, identification and recruitment

An online Delphi process was developed to engage experts and ascertain their opinions regarding indicators to be prioritized for appraising ASRH specifically for South Asia. Purposive sampling technique was used to identify and recruit experts for the survey. Experts were defined as:

1. Health researchers who published a peer-reviewed paper (any author position) on adolescent health, or sexual and reproductive health focused on South Asia.
2. Public health professionals working in program areas (regional/national experts, representatives of technical organizations, NGOs, public sector and health department/health ministry representatives) of adolescent health, sexual and reproductive health in South Asia.

Internet searches were conducted to identify experts according to the inclusion criteria. Email addresses were extracted from lists of authors or organization websites. Snowballing was also used for recruiting experts. For each round, an initial invitation was sent, and in case of no response, two reminders were sent. Experts who were invited for the rounds and did not respond after two reminders were not contacted for subsequent rounds.

### First round

The online survey was developed with Limesurvey (URL http://www.limesurvey.org). As explained above, 27 indicators on adolescent health from the WHO report were used for the first round. Experts were asked to rate the indicators on a Likert scale (5 = high relevance, 4 = relevant, 3 = moderate relevance, 2 = low relevance, and 1 = not relevant) based on relevance to ASRH in South Asia. Experts were also given the opportunity to recommend any other indicators that they deemed relevant during the first round. Indicators recommended by the experts were extracted through the online datasheet generated by the LimeSurvey. The list of indicators was compiled and duplicates were removed prior to rating in the second round.

### Second round

A list of 14 indicators, recommended by the experts in the first round, were rated in the second round. Results of the first round were also shared with the experts which included mean Likert scores along with the ranking of the 27 indicators.

### Third round

Experts were invited to a group discussion session to discuss the results using the specificity, measurability, achievability, relevance, and targeted (SMART) approach regarding the prioritized indicators. A discussion guide was prepared which included probes on indicator categories, ranking of indicators, survey data availability in South Asia, barriers in data collection on adolescent health indicators, and a detailed discussion on the results of the first and second rounds (Appendix A). Notes were taken during the group discussion sessions and results were compiled post-session. Results were sent to the experts who participated in the group discussion session for any additional feedback. Qualitative content analysis was used for coding and interpreting the qualitative data from the third round and the additional feedback sent by the experts.

### Delphi rounds and expert panel

**Figure 1.**
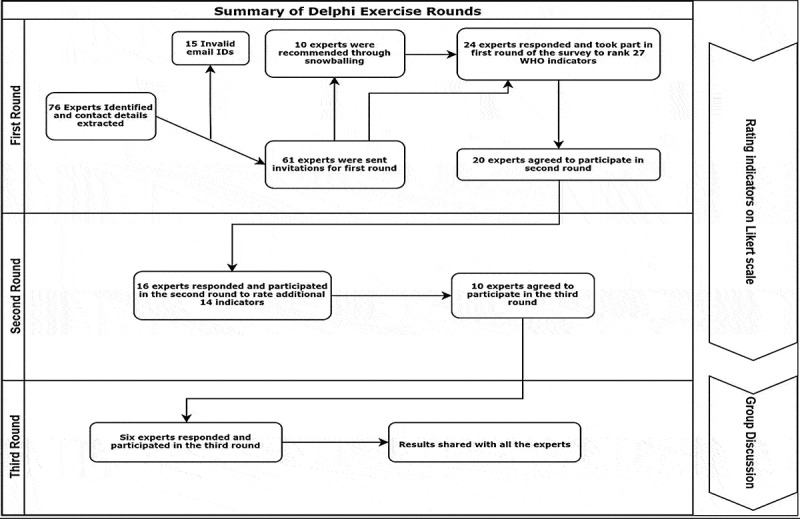
Summary of Delphi exercise rounds

**Figure 2.**
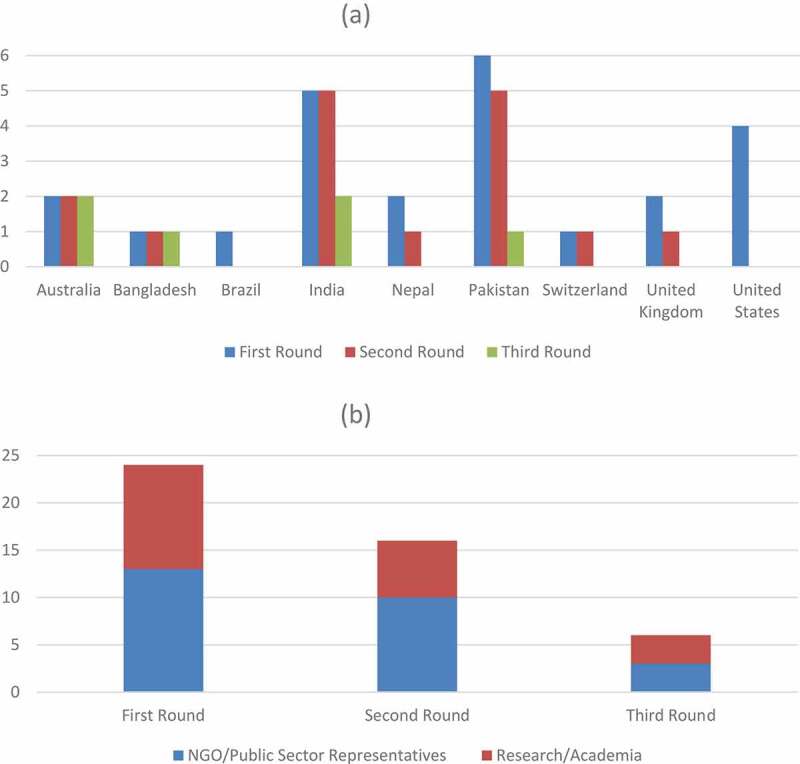
(a): Number of participating experts and their country of origin for all Delphi rounds. (b): Number of participating experts and their background based on inclusion criteria for all Delphi rounds

Seventy-six experts were identified as eligible to participate according to the specified criteria. Fifteen email IDs extracted through Internet search were invalid. As a result, 61 invitations were sent for the first round. Twenty-one experts responded for the first round (Figure 1). Ten additional experts were recommended through snowballing, out of which three experts responded. The participating experts were from Australia, Bangladesh, Brazil, India, Nepal, Pakistan, Switzerland, the UK, and the USA (Figure 2(a)). The experts had diverse professional backgrounds: academic institutes, research centers, United Nations agencies, public sector institutes, and non-governmental organizations (NGOs) (Figure 2(b)). Twenty experts agreed to participate in the second round out of which 16 responses were received. Six experts, of the 10 who agreed to participate in the third round, took part in the group discussion (Figure 1) . The Delphi was carried out between March and August 2019.

### Statistical analysis

The priority of indicators was determined using mean Likert scores whereas consensus among experts was assessed using Kendall’s Coefficient of Concordance (Kendall’s W) [33]. Kendall’s W is a non-parametric statistic used to assess agreement among raters. Its value ranges from 0 to 1 and the values were interpreted using the following cutoffs; 0.9 = unusually strong agreement, 0.7 = strong agreement, 0.5 = moderate agreement, 0.3 = weak agreement, and 0.1 = very weak agreement [33]. Kendall’s Tau (Tau-a and Tau-b) and Spearman’s Rank Correlation Coefficient were used to assess the strength of the relationship between the first-round indicator ranking and WHO indicator ranking [33,34]. The statistical analysis was conducted using Stata 14 (Stata, College Station, TX).

## Results

### Categorization and stratification of indicators

Categories developed by the WHO were used to group indicators for further statistical analysis [11,12]. The categories were further stratified based on whether the indicator was in the WHO list (first round 27 indicators) or recommended by experts (second round 14 indicators) (Table 1).

**Table 1.** Indicator category, ranking and descriptive statistics

Indicator	Indicator Category	Ranking based on mean Likert score	WHO Ranking (n/a for second round indicators)	N	Mean	Std. Err.	95% Conf. Interval	
Sexual abuse among adolescents	Violence	1	n/a	16	4.50	0.22	4.02	4.98
Adolescent fertility rate	Reproductive Health	2	7	24	4.46	0.17	4.11	4.81
Percentage of ever-married adolescents	Demographics	3	n/a	16	4.38	0.18	3.99	4.76
Abortion rates among adolescent girls (legal/illegal)	Reproductive Health	3	n/a	16	4.38	0.15	4.05	4.70
Adolescent maternal mortality ratio	Reproductive Health	5	6	24	4.33	0.21	3.89	4.78
Age at first birth among adolescents	Demographics	6	n/a	16	4.31	0.30	3.68	4.95
Literacy rate among adolescents	Demographics	6	n/a	16	4.31	0.27	3.74	4.89
Percentage of adolescents with improved knowledge and attitudes of Menstrual hygiene management practices	Reproductive Health	8	n/a	16	4.25	0.23	3.75	4.75
Demand for family planning satisfied with modern methods	Reproductive Health	9	14	24	4.12	0.26	3.59	4.66
Antenatal care coverage rate among adolescents	Reproductive Health	10	n/a	16	4.09	0.22	3.64	4.55
Percentage of deliveries by female adolescent attended by skilled birth attendant	Reproductive Health	11	n/a	16	4.06	0.21	3.61	4.52
Prevalence of intimate partner violence among adolescents	Violence	12	13	24	4.00	0.21	3.57	4.43
Prevalence of anemia among adolescents	Nutrition	13	9	24	3.96	0.22	3.50	4.42
Percentage of adolescent girls using hygienic sanitary pads	Reproductive Health	14	n/a	16	3.91	0.22	3.45	4.36
Prevalence of Reproductive tract infections among adolescents	Reproductive Health	14	n/a	16	3.91	0.27	3.32	4.49
Post natal care coverage rate among adolescents	Reproductive Health	16	n/a	16	3.84	0.29	3.22	4.47
Early initiation of sexual activity	Reproductive Health	17	11	24	3.83	0.25	3.33	4.34
Adolescent mortality rate from suicide	Mental health	18	4	24	3.79	0.23	3.31	4.27
Knowledge about HIV transmission among adolescents	Infectious/Communicable Diseases	18	18	24	3.79	0.23	3.33	4.26
Prevalence rate of suicide attempts among adolescents	Mental health	20	n/a	16	3.78	0.23	3.30	4.27
Condom use at most recent sex among adolescents with multiple sexual partnerships in past 12 months	Reproductive Health	21	12	24	3.75	0.28	3.18	4.32
Prevalence of underweight among adolescents	Nutrition	22	8	24	3.71	0.25	3.19	4.23
Current alcohol use among adolescents	Mental health	23	16	24	3.67	0.24	3.17	4.16
Adolescent mortality rate	Mortality	24	1	24	3.62	0.24	3.13	4.12
Smokeless tobacco prevalence among adolescents (pan, gutka, chalia, chewable tobacco products etc.)	Behavioral	25	n/a	16	3.59	0.30	2.95	4.23
Prevalence of insufficient physical activity among adolescents	Behavioral	26	17	24	3.58	0.25	3.06	4.11
Current tobacco use among adolescents	Behavioral	27	15	24	3.54	0.28	2.97	4.11
Prevalence of HIV infection among adolescents	Infectious/Communicable Diseases	28	21	24	3.50	0.23	3.03	3.97
Adolescents living with diagnosed HIV infection	Infectious/Communicable Diseases	28	23	24	3.50	0.22	3.05	3.95
HIV testing among adolescents	Infectious/Communicable Diseases	30	22	24	3.46	0.23	2.98	3.94
Antiretroviral therapy coverage of adolescents	Infectious/Communicable Diseases	31	24	24	3.42	0.22	2.97	3.86
Prevalence of overweight and obesity among adolescents	Nutrition	32	10	24	3.38	0.27	2.81	3.94
Parental connection with adolescents	Parental Connection and Regulation	32	19	24	3.38	0.22	2.93	3.82
New patients on antiretroviral therapy	Infectious/Communicable Diseases	34	25	24	3.33	0.21	2.89	3.78
HIV viral load suppression among adolescents	Infectious/Communicable Diseases	34	26	24	3.33	0.21	2.89	3.78
Human Pappilomavirus vaccination coverage among adolescents	Infectious/Communicable Diseases	36	n/a	16	3.22	0.31	2.55	3.89
Parental regulation of adolescents	Parental Connection and Regulation	37	20	24	3.21	0.20	2.80	3.62
Adolescent mortality rate from HIV/AIDS	Infectious/Communicable Diseases	38	3	24	3.17	0.27	2.61	3.72
Adolescent mortality rate from road traffic injuries	Mortality	39	2	24	3.12	0.28	2.54	3.71
Current cannabis use among adolescents	Mental health	40	27	24	3.00	0.28	2.43	3.57
Adolescent mortality rate from homicide	Violence	41	5	24	2.92	0.24	2.42	3.41

### Ranking and descriptive statistics

**Figure 3.**
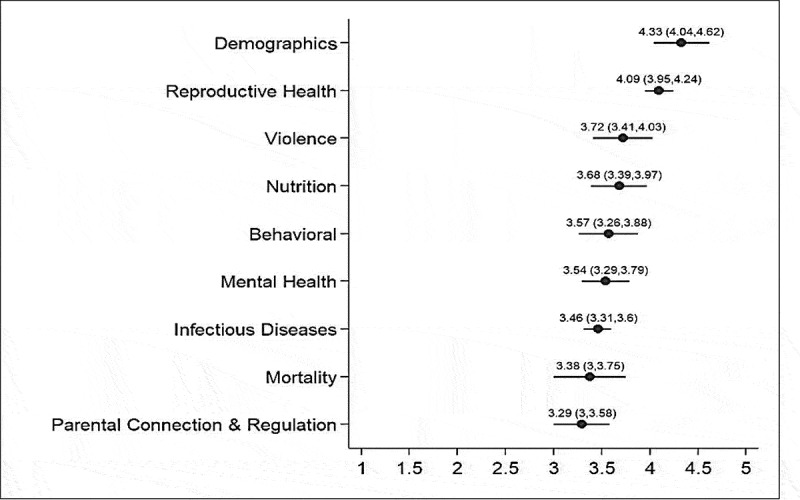
Indicator Category Ranking, mean scores and confidence interval plot

Table 1 shows indicator ranking, the WHO ranking (only for first-round indicators), mean Likert scores, standard error, and confidence intervals. Bootstrapping with 10,000 repetitions was used to generate the 95% confidence intervals. Twelve out of 14 indicators recommended by the experts in the second round were in the top 20 ranked indicators (Table 1). Indicator category ranking, mean scores, and confidence intervals are presented in Figure 3. Demographic and reproductive health indicator categories were rated highest and parental connection/regulation and mortality indicators were ranked lowest among the categories.

One possible concern with ranking first and second round indicators is that the number of experts reduced from 24 to 16 in the second round. Therefore, we tested the correlation in the ranking of the first-round indicators between the two groups of experts; 24 experts who participated in the first round and the 16 experts in the second round. The two rankings were compared using Kendall’s Tau (Tau-a and Tau-b) and Spearman’s Rank Correlation Coefficients (Appendix C). Kendall’s Tau-b suggests that the two groups of experts were 73.8% more likely to agree on the ranking of first-round indicators than to disagree (Appendix C).

### Need for regional indicator priorities

Using Kendall’s Tau-b, we compared the ranking of first-round indicators with the WHO’s ranking of indicators and observed that these two rankings were only 27% likely to agree on the importance of the first-round indicators. Similar results were obtained from Kendall’s Tau-a and Spearman’s Rank Correlation: 27% and 29%, respectively. Secondly, 12 out of the 14 second round indicators newly proposed by the participating experts were ranked in the top 20 (Table 1).

### Consensus among experts

The low estimated values of Kendall’s W suggest a very weak agreement among the experts for individual indicator ranking (Table 2). However, the agreement for indicator categories is substantially stronger (Table 2). Strong agreement was observed for the categories: Parental Connection/Regulation (W = 0.85), Behavioral (W = 0.71), Mortality (W = 0.69), Nutrition (W = 0.67), and Infectious Diseases (W = 0.67) (Table 2). Additionally, some of these categories included indicators from the first and second rounds (Table 1) which were further explored. Within these subcategories (first and second round indicators), the level of agreement increased even further (Table 2). Kendall’s W could not be calculated for second round mental health, violence and behavioral categories as these only had one indicator.

### Consensus versus relevance

**Figure 4.**
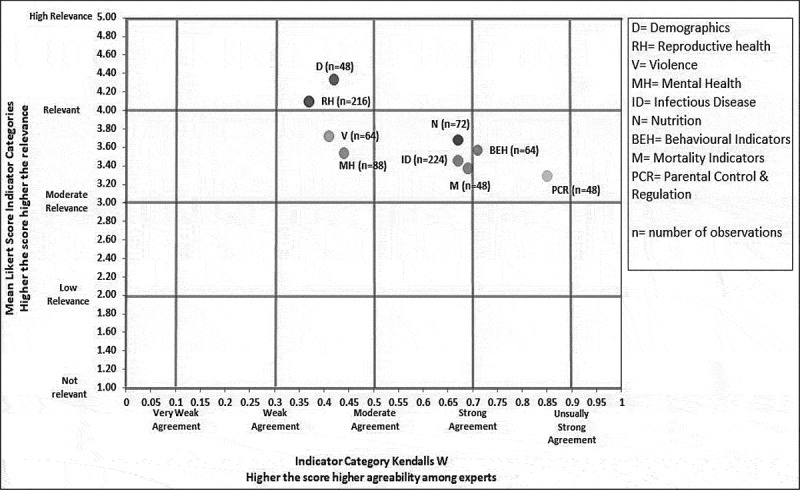
Assessing consensus (Kendall’s W) in contrast to relevance (Mean Likert scores) of indicator categories

Based on our indicator category Kendall’s W and mean scores, we observed that demographics, reproductive health, violence, and mental health ranked highest in terms of relevance but there was weak agreement among the experts for these categories (W < 0.5) (Figure 4). For nutrition, behavioral, infectious disease, and mortality indicator categories, the relevance was high to moderate and the experts showed a moderate agreement for these indicator categories (Figure 4). Experts had a strong agreement over the relatively low relevance of the parental connection/regulation category (Figure 4). Possible explanations for this variation in agreement among experts might be due to the different number of indicators in each category, as Kendall’s W is sensitive to the number of indicators.

### Round three group discussion

Six experts took part in the group discussion. The experts agreed that there is a need to develop specific regional priorities for indicators based on relevance, concurrent with our results. The experts also identified a lack of resources and dedicated trained human resources, non-existent data registries, cultural taboos, legal barriers, and lack of political support around sexual and reproductive health as barriers contributing to lack of data availability and health interventions for adolescents. The adolescent population is also not well represented in the current national-level health surveys in the region and efforts should be made to improve their inclusion and participation. Specifically, the experts from Bangladesh, India, and Pakistan highlighted that the early adolescent group (10–14 years) is not covered in national-level health surveys, whereas the middle/late adolescent group (15–19 years) is included. Experts also identified child marriage, inadequate access to contraception, and violence as some of the major issues faced especially by female adolescents in South Asia. Coded qualitative data are included as Appendix B.

## Discussion

The results of our study allow us to rank a wide-ranging list of indicators in terms of their relevance for assessing ASRH in South Asia. The list of indicators can be used to appraise the current situation and assess the determinants of sexual and reproductive health. It may also be useful for monitoring and evaluating specific ASRH programs and policies targeted at the adolescent population in South Asia.

Delphi rounds and results indicate that the process was successful in developing and promoting consensus on a comprehensive set of prioritized ASRH indicators. For this purpose, a multidisciplinary team of experts constituted the panel, and, through the Delphi rounds, they were able to prioritize a set of 41 indicators for appraising ASRH relevant to South Asia. The Delphi technique aided in systematically developing consensus and prioritization of the indicators. The Delphi process also made it easy to conduct the consensus exercise with an international expert panel using an online survey. Implementing the rounds online increased the ease and efficiency of the process, especially data collection, analysis, and communication across rounds with the expert panel. The experts were able to reach a consensus for multiple indicator categories. The prioritized list of 41 indicators will be able to provide a holistic approach for measuring ASRH in South Asia. The prioritized list constitutes indicators for health outcomes, lifestyle and health behaviors, healthcare services, and demographics which affects the adolescent population’s health in the region.

Based on our analysis, the demographics category was the highest-ranked which is in accordance with the literature, as demographics, in terms of age at first birth, literacy rate, and marital status among adolescents, have major implications for overall health outcomes of a population, in particular major adverse consequences on reproductive health outcomes and service utilization [23–25]. We established a strong to moderate expert consensus on most of the indicator categories. However, demographics and reproductive health indicator categories, although ranked the highest, had a relatively lower agreement among experts. There may be numerous reasons for the agreement among experts not being stronger; one possible explanation can be that both the categories had a wide range of indicators which could have contributed to the lower consensus. In contrast, the experts agreed on the lower relevance of parental connection/regulation and infectious disease categories in comparison to other categories. Eight indicators in the infectious disease category were related to HIV/AIDS, and experts seem to agree on its relatively lower ranking owing to the low prevalence of the disease in South Asia [35,36].

Understandably, owing to high fertility rates in South Asia [2], the adolescent fertility rate was ranked second among all indicators by the experts. Percentage of adolescents with improved knowledge and attitudes of menstrual hygiene management practices and adolescent girls using hygiene sanitary pads were ranked eighth and eleventh, respectively. There is a culture of silence around the menstrual health of girls due to societal stigma in South Asia [4–7,37]. Due to this, many girls do not understand their periods and lack awareness regarding menstrual hygiene which further contributes to the non-attendance of girls in schools. In the region, it is estimated that girls are absent for 20% of the school year owing to menstruation. Access to safe sanitary pads is limited for girls and women in the region because of the cost, lack of awareness, and societal stigma [4–7,37].

The experts ranked sexual abuse and abortion rates among adolescents as first and third, respectively, among all indicators. Almost half of the world’s children experience severe violence (physical, sexual, or emotional) and it is estimated that 64% of these children live in South Asia [38]. Almost half of unsafe abortions in the LMICs are occurring in Asia with sex-selective abortions and abortions due to unplanned pregnancy being quite common in South Asia [3,39,40]. Unfortunately, evidence-based information remains lacking on these issues, and, thus, obtaining reliable data remains a challenge. The WHO recommends that every child and adolescent have the right to comprehensive sexuality education (CSE), which includes age-appropriate information on various health topics including violence, sexual abuse, and planned parenthood [41,42]. Owing to community resistance, gaps in domestic legislation, non-existent national plans of action, inadequate law enforcement, and non-operative child protection systems in the region, adolescents have limited or no access to CSE and reproductive health services. This hampers the chances for substantial improvements in these aspects in the region [43–46].

As is frequently the case with the Delphi technique, the participation decreased for subsequent rounds which can be seen as one of the limitations of the study. Although eight to twelve participants are considered as an acceptable minimum for the Delphi technique, we tried to recruit as many experts who satisfied the inclusion criteria as possible to address the decrease in response rate for the subsequent rounds [29–32]. This also provided us with a wide-ranging panel of topic experts for the consensus exercise. The Delphi technique also allowed the experts to iterate their evaluation of the relevance of indicators anonymously limiting bias and peer influence. The Delphi technique relies on group consensus for decision among a group of experts. Prioritization of indicators was based on mean Likert score, but these scores might be influenced by individual expertise of the participating expert and can introduce bias based on individual preferences. Hence, consensus formation among the group of experts is an integral part of the Delphi process. For individual indicators, we found low consensus among the experts, this was per our expectations since the indicators deal with a wide variety of health issues and the experts were not expected to agree on the importance and relevance of all these indicators. For indicator categories, experts had strong to moderate consensus for most of the categories. Incorporating the group discussion session also allowed us to reflect the results from a wider regional perspective. As it was a qualitative round, the somewhat lower participation was not seen as a major limitation, and the session had no implications on the indicator ranking or consensus development among the expert panel.

However, the discussion session was highly informative. It emphasized that lack of resources, shortage of dedicated skilled human resources, non-existent data registries, and cultural restrictions affecting sexual and reproductive health significantly can contribute to a lack of data on adolescent health indicators. Non-inclusion of the early adolescent group and unmarried females adds to the unavailability of indicator data in national representative surveys. These barriers and limitations should be further investigated to explore possible potential enablers and explanations to improve data availability on adolescent health indicators.

When comparing the WHO adolescent health indicator ranking to the first-round indicator ranking, we found a low correlation between the two rankings. Twelve out of the 14 second round indicators, recommended by the expert panel, were also ranked in the top 20. Even though the WHO’s ranking was developed for adolescent health and we focus specifically on ASRH, low correlation of the rankings and the additional second-round indicators with high mean Likert scores support the need for regional prioritization of indicators concurrent with the WHO and expert recommendations [11,12]. DHS and MICS are currently in use a universal survey tool for collecting health indicator data across the LMICs [21,22]. These tools allow monitoring, appraisal, and comparison of health indicators across countries and regions. Although the regional indicator priorities might provide rich information on the health patterns and trends, they pose a challenge for comparison across different countries and regions at the same time.

This is one of the first studies to employ the Delphi technique for prioritizing ASRH indicators for South Asia. Using the online platform, engaging a diverse group of experts, and developing a comprehensive list of key indicators for appraising ASRH, are some of the major strengths of the study. Also, the Delphi rounds were systematic and well-structured which complements the validity of the findings of this study. Further studies are required to explore whether tailored region-specific surveys can provide better insights on population health, for example, regarding their potential policy impact.

## Conclusion

This study explored the opinions of topic experts from diverse backgrounds on the relevance of key indicators for appraising ASRH in South Asia. Using the Delphi method, we determined a prioritized list of ASRH indicators, based on expert panel consensus and relevance, for South Asia. Our statistical analysis of the first and second round highlighted the need for developing region-specific indicator prioritization. Additionally, the discussion round highlighted the barriers and limitations to improved data availability on adolescent health indicators in South Asia.

## Paper context

Comprehensive sets of health indicators support in formulating evidence-based policies. Indicators should be developed and prioritized based on consensus and regional relevance. This is one of the first studies to employ the Delphi technique for prioritizing adolescent sexual and reproductive health (ASRH) indicators for South Asia. Using the online platform, engaging a diverse group of experts, we developed a list of key indicators for appraising ASRH for South Asia.

## Appendix A. Focus group discussion guide for Round 3

**Welcome**

Introduce yourself and go around the group participants for a brief introduction. (5 minutes)

**Points of discussion**

•A brief summary of the survey phase 1 and 2 (5 minutes)

•Discussion on the results of phase 2 (ranking of the indicators, review of the result sheet) (10–15 minutes)

•Explain the process of result compilation (5 minutes)

•Start the discussion on the categories of the indicators (5 minutes)

•Following the SMART approach, proceed with the discussion on each category of indicators (5–7 minutes each category)

•Discussion on strategies to improve adolescent health inclusion in national level health and demographic surveys (5–10 minutes)

•Recommendations on developing indicators and regional priorities.

•Final remarks and concluding the session (5 minutes)

The discussion will be carried out on 9 categories using the SMART approach.

**Table:**

S.No.	Category	Phase 1 Indicator	Phase 2 Indicator
1	Nutrition	Prevalence of anemia among adolescents Prevalence of underweight among adolescents Prevalence of overweight and obesity among adolescents	
2	Mortality	Adolescent mortality rate Adolescent mortality rate from road traffic injuries	
3	Reproductive Health	Adolescent fertility rate Demand for family planning satisfied with modern methods Early initiation of sexual activity Condom use at most recent sex among adolescents with multiple sexual partnerships in past 12 months Adolescent maternal mortality ratio	Percentage of adolescents with improved knowledge and attitudes of Menstrual hygiene management practices Antenatal care coverage rate among adolescents Percentage of deliveries by female adolescent attended by skilled birth attendant Post natal care coverage rate among adolescents Abortion rates among adolescent girls (legal/illegal) Percentage of adolescent girls using hygienic sanitary pads
4	Infectious Diseases	Knowledge about HIV transmission among adolescents Adolescent mortality rate from HIV/AIDS New patients on antiretroviral therapy HIV viral load suppression among adolescents Prevalence of HIV infection among adolescents Adolescents living with diagnosed HIV infection HIV testing among adolescents Antiretroviral therapy coverage of adolescents	Prevalence of Reproductive tract infections among adolescents Human Papillomavirus vaccination coverage among adolescents
5	Behavioral	Prevalence of insufficient physical activity among adolescents Current tobacco use among adolescents	Smokeless tobacco prevalence among adolescents (pan, gutka, chalia, chewable tobacco products etc.)
6	Violence	Prevalence of intimate partner violence among adolescents Adolescent mortality rate from homicide	Sexual abuse among adolescents
7	Parental Connection and Regulation	Parental connection with adolescents Parental regulation of adolescents	
8	Demographic		Percentage of ever-married adolescents Age at first birth among adolescents Age at first birth among adolescents
9	Mental health	Current alcohol use among adolescents Current cannabis use among adolescents Adolescent mortality rate from suicide	Prevalence rate of suicide attempts among adolescents

**Probes for Discussion**

**Table:**

Specific	Measurable	Achievable	Relevance	Targeted
Is it clear what is being measured? Is there a need to have specific indicators for ARH regional specific? Will it improve the information in context to specificity?	Definition availability? Numerator/denominator? Is it possible to collect the data? Segregation of data?	Is the indicator routinely collected at a national level? Is it possible to collect data on these indicators?	Scoring conducted on relevance in round 1 and 2. Impressions on the results.	Are the indicators targeting adolescent’s sexual and reproductive health? Will the targeted approach has the potential to improve the data collected?

**Achievable**

**What are the possible strategies to make recruitment and inclusion of adolescents in surveys on ARH in south Asian countries?**

•How to tackle cultural sensitivities?

•How to recruit?

•Where to recruit?

•Inclusion of unmarried individuals?

•Can combined sessions with adolescents and gatekeepers (parents, teachers etc.) be a good strategy to get information on SRH?

**Indicator Prioritization**

•DHS has a universal list of indicators for all the countries they have surveys in, is there a need to have indicators specific for regions?

•Do we need indicator list on the basis of age groups, or specific for reproductive health, mental health, nutrition, etc. or both?

•Why?

•Do we need to have regional indicators for other regions as well?

**Recommendations for Indicator Prioritization**

– – – – – – – – – – – – – – – – – – – – – – – – – – – – – – – – – – – – – – – – – – – – – – – – – – – – – – – – – – – – – – – – – – – – – – – – – – – – – – – – – – – – – – – – – – – – – – – – – – – – – – – – – – – – – – – – – – – – – – – – – – – – – – – – – – – – – – – – – – – – – – – – – – – – – – – – – – – – – – – – – – – – – – – – – – – – – – – – – – – – – – – – – – – – – – – – – – – – – – – – – – – – – – – – – – – – – – – – – – – – – – – – – – – – – – – – – – – – – – – – – – – – – – – – – – – – – – – – – – – – – – – – – – – – – – – – – – – – – – – – – – – – – – – – – – – – – – – – – – – – – – – – – – – – – – – – – – – – – – – – – – – – – – – – – – – – – – – – – – – – – – – – – – – – – – – – – – – – – – – – – – – – – – – – – – – – – – – – – – – – – – – – – – – – – – – – – – – – – – – – – – – – – – – – – – – – – – – – – – – – – – – – – – – – – – – – – – – – – – – – – – – – – – – –

**Concluding the Session**

Thank you so much for coming and sharing your thoughts and opinions with us. If you have additional information that you did not get to say in the focus group, please feel free to write it and send it to us through email.

## Appendix B. Quotation report ‒ Grouped by: Codes

**○ Child marriage**

‘The precedence of customary law in rural areas around the minimum age of marriage is a further factor’

**○ Contraception**

‘Adequate provisions of modern forms of contraception to adolescents’

‘Legal barriers e.g. the inability to prescribe contraception to sexually active girls under the age of 18 who are not married, is a big problem’

‘Reducing stigma around provision of contraception to unmarried adolescents’

**○ Difficulty in determining age and non-inclusion of adolescents in surveys**

‘Determination of age and inclusion of early age (adolescents) is becoming increasingly problematic in some south Asian settings’

‘No policy level focus and adolescent not identified as a specific population with specific needs’

**○ Digital interventions**

‘Possible mode (intervention) can be audio visual aid delivered in schools. Other can be use of digital technology for the health care provider’

‘Counselling and education via media and internet through invisible means should be the predominant mode in current scenario with enough advertisement so that the target audience is aware of such means. These mass-media communications should be short, smart, and appealing to overcome shyness and boredom’

‘This should be followed by the option of face-to-face counselling and interventions which adolescents can avail easily. Here, the providers have to be carefully trained in adolescent friendly behaviours. In order to use above strategy, media and internet use among adolescents will be needed’

**○ Education**

‘And gender norms that disempower girls and young women, together with lack of educational opportunity are further factors’

‘Socio-cultural norms that exclude girls from education, in particular life skills based education for adolescents’

‘There are cultural inhibitions among women to access health institutions for information, services, etc.’

‘Through education and counselling in schools and community’

‘Health interventions in adolescents should be couple with other interventions like skill improvement, vocational training, career counselling … … ’

‘Lack of age appropriate health and family planning education and health services ….’

‘Counselling and education via media and internet through invisible means should be the predominant mode in current scenario with enough advertisement so that the target audience is aware of such means. These mass-media communications should be short, smart and appealing to overcome shyness and boredom’

‘This should be followed by the option of face-to-face counselling and interventions which adolescents can avail easily. Here, the providers have to be carefully trained in adolescent friendly behaviours. In order to use above strategy, media and internet use among adolescents will be needed’

**○ Health Services and Provider’s**

‘Major barriers are the availability and the accessibility of health services to adolescents, lack of skills amongst the health care providers, lack of social and political support for addressing adolescent health’

‘Sensitization of staff in public health facilities through group discussions and interactive sessions’

‘When adolescents reach a certain age, do they have routine check-ups with their doctors regarding their overall and reproductive health’

‘Through public awareness, seeping that into educational resources, schools, hospitals, birthing clinics. Also nurses and doctors need training on how to convey and portray the message of reproductive health positively in areas where due to societal and cultural barriers, the message may not be taken in seriously and for data collection’

‘This should be followed by the option of face-to-face counselling and interventions which adolescents can avail easily. Here, the providers have to be carefully trained in adolescent friendly behaviours. In order to use above strategy, media and internet use among adolescents will be needed’

**○ Inclusion of adolescent population**

‘It will vary between states, divisions and countries but obviously include primary health care, community-based services e.g. girls clubs, school platforms, digital media and probably all of the above’

‘Ecological model with use of the technologies could be effective strategy!’

‘In South Asian countries, possible modes of delivery for adolescent reproductive health improvement include brochures, pamphlets in local languages as well as counselling in schools, health facilities and at community centres, homes, by lady health workers. Staff may include teachers, health workers, school medical officers’

‘No policy level focus and adolescent not identified as a specific population with specific needs’

‘Through education and counselling in schools and community’

‘Possible mode can be audio visual aid delivered in schools. Other can be use of digital technology for the health care provider’

‘Counselling, self-help groups, focus group discussions at community level to access the most vulnerable populations’

‘Sensitization of staff in public health facilities through group discussions and interactive sessions’

‘Lack of age appropriate health and family planning education and health services add to non-inclusion’

**○ Lack of data registries**

‘Many of these indicators are under pressure because of the lack of vital registration systems’

‘I’ve already flagged the lack of vital registration systems is a further problem in terms of implementing legislation’

‘No policy level focus and adolescent not identified as a specific population with specific needs’

**○ Lack of political support**

‘Major barriers are the availability and the accessibility of health services to adolescents, lack of skills amongst the health care providers, lack of social and political support for addressing adolescent health’

‘Poor implementation of laws and policies’

**○ Legal barriers**

‘Legal barriers e.g. the inability to prescribe contraception to sexually active girls under the age of 18 who are not married, is a big problem’

‘I’ve already flagged the lack of vital registration systems is a further problem in terms of implementing legislation’

‘The precedence of customary law in rural areas around the minimum age of marriage is a further factor’

‘Major barriers are the availability and the accessibility of health services to adolescents, lack of skills amongst the health care providers, lack of social and political support for addressing adolescent health’

‘Poor implementation of laws and policies’

**○ Nutritional issues**

In some parts of South Asia, stunting and nutrition remain a major problem”

**○ Social norms**

‘Social norms around early marriage remain a major barrier’

‘And gender norms that disempower girls and young women, together with lack of educational opportunity are further factors’

‘Illiteracy, gender, and cultural related barriers still exist in communities’

‘Prejudices related to contraception before first child contributes to lack of family planning’

‘Socio-cultural norms that exclude girls from education, in particular life-skills based education for adolescents’

‘Cultural inhibitions among women to access health institutions for information, services, etc.’

‘Major barriers are the availability and the accessibility of health services to adolescents, lack of skills amongst the health care providers, lack of social and political support for addressing adolescent health’

‘Reducing stigma around provision of contraception to unmarried adolescents’

‘The stigma and marginalization that is attached to it. The topic is not openly shared and talked about because of cultural and societal barriers’

‘Through public awareness, seeping that into educational resources, schools, hospitals, birthing clinics. Also nurses and doctors need training on how to convey and portray the message of reproductive health positively in areas where due to societal and cultural barriers, the message may not be taken in seriously’

**○ Specificity**

‘Think many of these indicators are context specific and impact, prevalence etc. will differ between countries (South Asia)’

**○ Violence**

‘Violence against adolescent like eve teasing, sexual harassment is a concern’

## Appendix C.Tau-a, Tau-b and Spearman’s coefficient are reported for the rankings, for all experts (n = 24, First round) and experts present for both rounds (n = 16, Second round). Bootstrapping with 10,000 repetitions was employed to generate the 95% confidence intervals

**Table:**

Correlation Coefficient	Statistic	Bootstrap Std. Err.	Z	Normal based [95% Conf. Interval]	
Tau-a	0.7236***	0.0657	11.01	0.5948	0.8525
Tau-b	0.7384***	0.0688	10.73	0.6036	0.8732
Spearman’s	0.8966***	0.0484	18.54	0.8018	0.9914

Significance test is reported as *** p < 0.01, ** p < 0.05, * p < 0.10. The values of these statistics range from −1 to 1. However, Spearman’s statistic requires a monotone relationship between the two variables while Kendall’s Taus do not have any such requirement. On the other hand, Kendall’s Tau-a does not make any adjustment for ties whereas Tau-b makes appropriate adjustments. As such, Kendall’s Tau-b is the more relevant statistic for our scenario.
